# Predictions of melatonin suppression during the early biological night and their implications for residential light exposures prior to sleeping

**DOI:** 10.1038/s41598-020-70619-5

**Published:** 2020-08-24

**Authors:** Mark S. Rea, Rohan Nagare, Mariana G. Figueiro

**Affiliations:** Lighting Research Center, Rensselaer Polytechnic Institute, 21 Union Street, Troy, NY 12180-3352 USA

**Keywords:** Circadian mechanisms, Circadian regulation

## Abstract

The magnitude of nocturnal melatonin suppression depends upon the spectrum, amount, and duration of light exposure. The functional relationship between melatonin suppression and the light spectrum and amount have been previously described. Only one duration-dependent parameter was needed to extend this functional relationship to predict nocturnal melatonin suppression during the early biological night from a variety of published studies. Those predictions suggest that ambient lighting commonly found in North American homes will not suppress melatonin for durations up to 3 h, whereas extended use of self-luminous displays in the home prior to sleep can.

## Introduction

The circadian system is perhaps one of the most important non-visual systems affected by retinal light exposure. The retinohypothalamic tract (RHT) is the direct neural pathway from the retina to the master biological clock, the suprachiasmatic nuclei (SCN) in the hypothalamus. It is now known that the spectral, temporal, spatial, and absolute sensitivity characteristics of the RHT neural channel stimulating the SCN are quite different from those exhibited by the optic nerve leading to visual functioning by the thalamus and visual cortex. This is true even though all retinal photoreceptors, including the intrinsically photosensitive retina ganglion cells (ipRGCs), participate in the various phototransduction processes for visual and non-visual systems^1–6^. To quantify light as a stimulus for the circadian system, it is necessary to develop a functional relationship between optical radiation incident on the retina and the spectral, temporal, and absolute responses of the SCN. Toward that end, classic psychophysical methods can be used^7^.

## Defining light: the photopic luminous efficiency function, V(λ)

**Figure 1:**
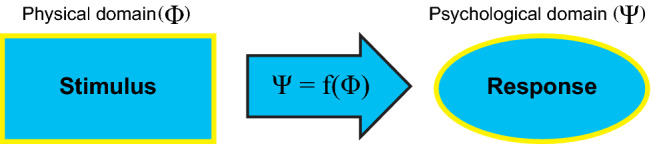
Psychophysics, first defined by Gustav Fechner in his treatise *Elements of Psychophysics* in 1,860^7^, is the establishment of functional relationships between the physical domain (Φ), the measured stimuli, and the psychological domain (Ψ), the measured behavioral responses.

Psychophysics is a technique used to derive functional relationships between physical (Φ) stimuli and psychological (Ψ) responses (Fig. 1). Like physical quantities, these psychological responses are always in the form of measurable behavior, such as reaction times, subjective judgments, or nocturnal melatonin suppression.

Radiometry is the physical measurement of optical radiation. A wide variety of behavioral responses can be measured for the very same optical radiation incident on the retina in psychophysical experiments. For example, an exposure to monochromatic wavelength can evoke a reaction time, a subjective report of apparent brightness, a reported hue sensation, or suppression of melatonin synthesis. Each of those behavioral responses will have a different functional relationship to the monochromatic wavelength exposure.

The goal of one set of psychophysical experiments conducted in the early part of the twentieth century was to define *light* as a physical quantity for the emerging lighting industry by establishing a functional relationship between the radiant power of monochromatic wavelengths to the relative sensitivity of the human visual system to those wavelengths. Since sensitivity cannot be measured directly, the basic rationale for these experiments was to measure psychological judgements of equality for two monochromatic wavelengths and then piece together a relative sensitivity function from all wavelengths that had been judged as equal. Specifically, if any two monochromatic wavelengths were reported to be equal, it was assumed that both would have been stimulated by the same physical amount of *light*.

**Figure 2:**
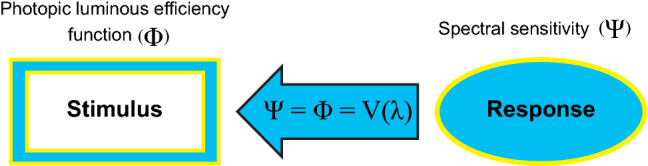
Once a psychophysical relationship has been established between the stimulus and the response, the behavioral response from the psychological domain (Ψ) becomes a measure of the stimulus in the physical domain (Φ). Early studies of the spectral sensitivity of humans to optical radiation became the foundation for the photopic luminous efficiency function, V(λ), which is now used to characterize light as a stimulus for human vision.

Two equality techniques were used. One was based upon direct comparisons of the perceived brightness of two wavelengths while the other was based upon the disappearance of flicker for two rapidly oscillating wavelengths. For both techniques the radiant power of a reference wavelength was fixed and visually compared to a test wavelength with variable radiant power. The radiant power of the test was varied until the two wavelengths were judged equal (i.e., equal apparent brightness or no apparent flicker). The relative amount of radiant power needed by the test wavelength to be perceived as equal to the radiant power of the reference wavelength was then measured. Both of these equality methods were applied for pairs of wavelengths across the spectrum. From these psychophysically determined equal wavelengths, the wavelength requiring the least amount of radiant power to be equal to the others was at 555 nm. This was the wavelength to which people were most sensitive and therefore the peak of the spectral sensitivity function that would define light for the Commission Internationale de l'Éclairage (CIE). Once the CIE adopted this, so-called photopic luminous efficiency function, V(λ), in 1924 to define light, a system of measuring optical radiation as a visual *stimulus* was possible. Thus, through psychophysics one aspect of the psychological domain (Ψ), a spectral sensitivity function, became a member of the physical (Φ) domain, the photopic luminous efficiency function (Fig. 2).

**Figure 3:**
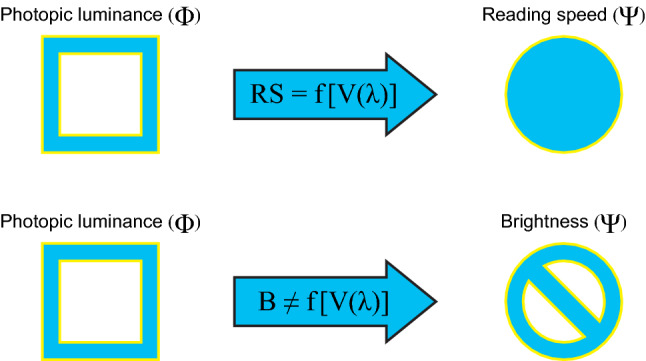
The photopic luminous efficiency function, V(λ), is excellent at characterizing the visual stimulus for reading materials, but not for subjective brightness. Therefore, light stimuli defined in terms of V(λ) in the physical domain (Φ) have only limited ability for establishing psychophysical relationships to other responses in the psychological domain (Ψ). B, brightness; RS, reading speed.

This 1924 system of photometry has been an extremely useful simplification for the lighting industry because the spectral characteristics of the (implicit) visual stimulus can be described in terms of a single quantity (e.g., luminance) without consideration of the spectral power distribution of the light source. Thus, intensity distributions (luminous intensity) and recommended light levels (illuminance) can all been defined in terms of a system of photometry based upon V(λ). Indeed, for many visual tasks that are processed by the fovea, like reading or on-axis detection, V(λ) is an excellent rectifying measure of the spectral characteristics of the visual stimulus^8^. In graphical terms, the psychophysically determined functional relationship can be shown as a plot of the physical quantity represented on the abscissa, in appropriate units (e.g., wavelength in nm), and the psychological response on the ordinate (e.g., reciprocal of the radiant watts seen as equal). For other behavioral responses like subjective brightness, however, V(λ) is not a suitable rectifying measure of the spectral characteristics of the visual stimulus (Fig. 3)^9^. Short wavelengths, discounted by V(λ), strongly affect brightness response.

It should be noted that specification of the spectral characteristics of the visual stimulus does not represent a full specification of the stimulus. Additional psychophysical experiments need to be performed to establish a functional relationship between a set of physically measured stimuli and a psychological response. For example, to predict on-axis detection, the solid angle of the visual target and its contrast as well as the absolute luminance of the target background must be known. All of these stimulus characteristics provide input into the relative visual performance (RVP) model, which has been validated for reading speed and for a variety of other on-axis responses^10–14^. Once the functional relationship between the physical stimulus and psychological response is established, however, the response metric (e.g., RVP) becomes part of the physical domain (Φ) even if the physical stimulus is complex.

## Defining circadian light and circadian stimulus

Given the conventional measures of light based upon the photopic luminous efficiency function, V(λ), do not appropriately represent the circadian system’s response to different wavelengths, a new spectral sensitivity function was needed. *Circadian light* (CL_A_) was derived, in part, from binocular experiments^15,16^ using monochromatic light-induced nocturnal melatonin suppression as the outcome measure^17,18^. The pineal gland synthesizes melatonin at night and because there is only one major light-sensitive pathway from the SCN to the pineal gland^19,20^, nocturnal melatonin suppression is an ideal way to measure the otherwise unreachable response of the human SCN to different spectra and amounts of retinal light exposure.

Circadian light (CL_A_) was developed to be analogous to V(λ) but, as the name implies, to define *light* for the circadian system. Like V(λ), CL_A_ was based upon a particular type of psychophysical experiment, specifically, light-dependent attenuation of melatonin synthesis by the pineal gland at night. Using a constant criterion methodology to establish equality of response, it was possible to quantify the relative radiant power needed to suppress nocturnal melatonin synthesis by monochromatic light sources^15,16^. Although these data provided an important starting point for the development of CL_A_, subsequent experiments with polychromatic sources showed that the response of the circadian system to broad-band spectra could not be predicted from the spectral sensitivity data obtained from monochromatic sources^21–23^. Specifically, under some conditions adding more light reduced the circadian system response; this is known as subadditivity. Consequently, a more complicated formulation of circadian light had to be developed to describe the spectral sensitivity of the circadian system to any spectral power distribution, monochromatic or polychromatic. This more complete formulation then, just like V(λ), moves CL_A_ from the psychological domain (Ψ) to the physical domain (Φ).

As already discussed, defining circadian light is not a full specification of the stimulus for the circadian system. A more complete specification would also include the operating range of the circadian system from threshold to saturation in addition to its spectral sensitivity. The metric *circadian stimulus* (CS) was developed for this purpose. As with CL_A_, psychophysical experiments using nocturnal melatonin suppression informed the development of CS. The absolute sensitivity of the circadian system to luminous stimuli, quantified in terms of CL_A_, was functionally described by CS for a 1-h exposure duration^24^. Thus, CS moved from the psychological domain (Ψ) to the physical domain (Φ) for specifying luminous stimuli for the circadian system.

CS is not a complete specification of the luminous stimulus, however. The duration of exposure is also important for describing the circadian system’s response to optical radiation on the retina. The purpose of the present paper was to determine how the duration of exposure could be added to the CL_A_ and CS formulations to predict nocturnal melatonin suppression during the early biological night. Specifically, melatonin suppression data for different binocular exposure durations recently reported by Nagare et al.^25^ were used to develop a more complete specification of the circadian stimulus.

## Methods

### Current CS model

According to the circadian phototransduction model (Eqs. 1 and 2) proposed by Rea et al.^17,18,24^, CL_A_ represents the spectral sensitivity of the SCN to light and CS represents its absolute sensitivity. Although the CL_A_ and CS formulations were based upon nocturnal melatonin suppression following a 1-h light exposure, the model is intended to represent the circadian system’s instantaneous response to light exposures. To accurately predict the amount of nocturnal melatonin suppression for light exposures other than 1 h, however, the duration of light exposure must also be known.

1 $$CL_{A} = 1,548\left\{ {\begin{array}{*{20}l} {\int {Mc_{\lambda } E_{\lambda } d\lambda + a_{b - y} \left( {\int {\frac{{S_{\lambda } }}{{mp_{\lambda } }}E_{\lambda } {\text{d}}\lambda - k\int {\frac{{V_{\lambda } }}{{mp_{\lambda } }}E_{\lambda } {\text{d}}\lambda } } } \right)} - a_{rod} \left( {1 - e^{{\frac{{ - \int {V_{\lambda }^{{\prime}} E_{\lambda } {\text{d}}\lambda } }}{RodSat}}} } \right),} \hfill & {b - y > 0} \hfill \\ {\int {Mc_{\lambda } E_{\lambda } d\lambda ,} } \hfill & {b - y \le 0} \hfill \\ \end{array} } \right.$$

2 $$CS=0.7-\frac{0.7}{1+{\left(\frac{C{L}_{A}}{355.7}\right)}^{1.1026}}$$

where,

$$b-y=\int \frac{{S}_{\lambda }}{{mp}_{\lambda }}{E}_{\lambda }\mathrm{d}\lambda -0.2616\int \frac{{V}_{\lambda }}{{mp}_{\lambda }}{E}_{\lambda }\mathrm{d}\lambda $$

*CL*_*A*_: Circadian light, ${E}_{\lambda }:$ light source spectral irradiance, *CS*: Circadian stimulus, ${Mc}_{\lambda }$: melanopsin sensitivity (corrected for crystalline lens transmittance), *k* = 0.2616, ${S}_{\lambda }:$ S-cone fundamental, ${a}_{b-y}=0.7$, ${mp}_{\lambda }$: macular pigment transmittance, ${a}_{rod}=3.3$, ${V}_{\lambda }:$ photopic luminous efficiency function, $RodSat=6.5 W {m}^{-2}$, ${V{^{\prime}}}_{\lambda }:$ scotopic luminous efficiency function.

### Test dataset

The data recently published by Nagare et al.^25^ (Table 1) were used to determine whether the CS formulation could be supplemented with an exposure duration term to predict nocturnal melatonin suppression in the early biological night. Nagare and colleagues measured light-induced nocturnal melatonin suppression in healthy adults and adolescents following binocular exposure to a wide range of light levels (40–1,000 lx), two white-light spectra (2,700 K and 6,500 K), and extended nighttime light exposure durations (0.5–3.0 h). (The α-opic irradiances (μW cm^−2^) for the lighting interventions, following the SI-compliant approach recommended by the CIE^26^ are provided in the original manuscript.) Statistical analysis showed that the main effect of participant age was not significant, nor were the two-way interactions between age and light level, spectrum or duration (*p* > 0.05), so the melatonin suppression values for the two age groups were averaged together for each combination of the four light levels, two spectra, and six durations.

**Table 1:** Nocturnal melatonin suppression, in percent, from Nagare et al.^25^ together with the average CS and CL_A_ levels recorded by participants’ Daysimeters, light-weight, head-mounted devices developed by Bierman et al.^27^ to measure individual light exposures during the experiment.

Spectrum	Amount		Nocturnal melatonin suppression (%) by exposure duration (h)					
	CS	CL_A_	0.5 h	1.0 h	1.5 h	2.0 h	2.5 h	3.0 h
2,700 K	0.06	41.6	− 1	2	2	2	3	6
	0.11	77.5	2	7	7	5	11	13
	0.27	233.2	12	17	21	22	25	25
	0.45	606.2	26	39	45	50	53	58
6,500 K	0.06	41.6	2	5	0	− 1	5	8
	0.11	77.5	3	7	11	12	17	19
	0.28	246.3	19	28	33	37	40	44
	0.46	641.7	26	43	51	57	61	65

Based upon the specific experimental setup as described in Nagare et al.^25^, each study participant either operated an electronic device (e.g., filtered laptop or smartphone) or read a physical book on the desk. Retinal light exposures for each participant were monitored using a Daysimeter (Model 12, Lighting Research Center, Troy, NY) mounted on a lensless eyeglasses frame throughout the 3-h light exposure. A simple exercise was conducted wherein photometric measurements were taken to address any systematic offset in effective corneal stimulus due to misalignment between the participant line of sight and the orientation of the Daysimeter sensor. The exercise revealed that the effective light level at the participants’ eye was reduced by an average of 23.4% (SD 2.5) and 16.8% (SD 3.2) while operating a smart phone or a laptop, respectively, and reduced by 24.3% (SD 3.7) when reading a book. Therefore, the CL_A_ values in Table 1 were multiplied by a factor of 0.797 (aggregate), consequently reducing the effective CS levels for modeling purposes. Post hoc statistical analysis showed that the measured nocturnal melatonin suppression from Nagare et al.^25^ and effective CS did not differ significantly (*p* > 0.05; Supplementary Table S1).

### Mathematical modeling

The statistical analyses reported in the original publication revealed that there was no significant interaction between light spectrum and duration (*p* > 0.05), suggesting that over the range of conditions employed by Nagare et al.^25^ the spectral sensitivity of the circadian system did not change. It should be true then that the form of the sigmoidal four-parameter logistic function in Eq. 2 would remain unchanged and the basic function simply would be shifted along the log CL_A_ abscissa as a function of exposure duration. Guided by parsimony and the assumption that spectral sensitivity did not change over 3 h, it was assumed for modeling purposes that only the half-saturation constant in Eq. 2 (i.e., CL_A_ = 355.7) would systematically change as a function of exposure duration. Thus, Eq. 2 was modified slightly (Eq. 3), whereby *q* was the only variable.

3 $$CS=0.7*\left[1-\frac{1}{1+{\left(\frac{{CL}_{A}}{q}\right)}^{1.1026}}\right]$$

## Results

### Test dataset optimization

**Figure 4:**
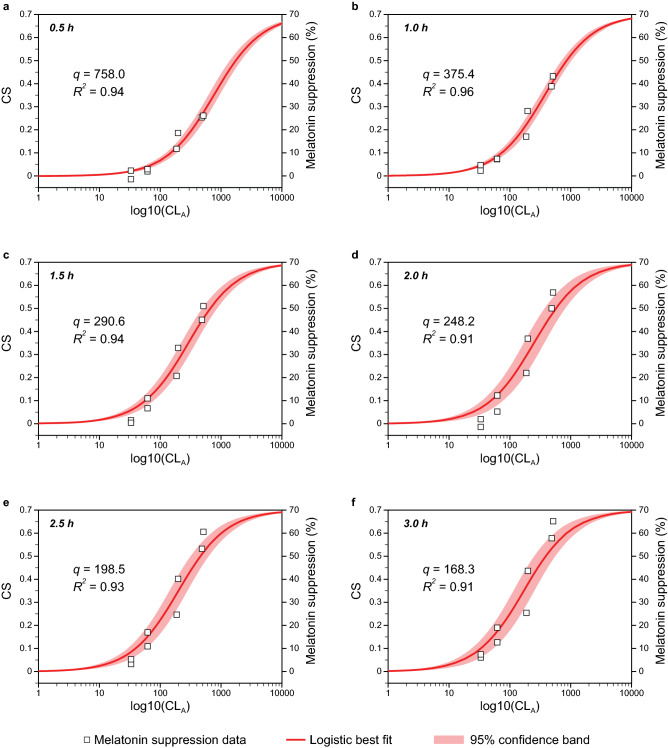
Optimized logistic functions from Eq. 3 relating nocturnal melatonin suppression to log CL_A_ for different exposure durations (0.5–3 h [**a**–**f**, respectively]) together with a summary of the inferential statistics. The only free parameter was *q*, the half-saturation value for each exposure duration.

Best fitting logistic functions based upon Eq. 3 and developed using curve fitting software OriginPro 2020 (OriginLab Corporation, Northampton, MA) for the six different exposure durations from Nagare et al.^25^ are shown in Fig. 4. All the curves fit the melatonin suppression data significantly (*p* < 0.001) and the goodness of fit as assessed by the coefficient of determination (*R*^2^) was always greater than 0.90. The optimized half-saturation constant (*q*) values ranged from CL_A_ = 168.3 for the 3.0-h test dataset to CL_A_ = 758.0 for the 0.5-h test dataset.

The free parameter, *q*, in Eq. 3 can be considered as a light exposure (amount × duration) term, where

4 $$q={q}_{m}*{t}^{r}$$

and *t* is the duration of light exposure, in hours, *q*_*m*_ is the amount of measured light, in terms of CL_A_, producing half saturation and, to avoid any assumption of reciprocity between the amount of exposure and the duration of exposure, *r* is a free parameter. In the original model by Rea and colleagues, *q* = 355.7 for a 1-h exposure duration.

**Figure 5:**
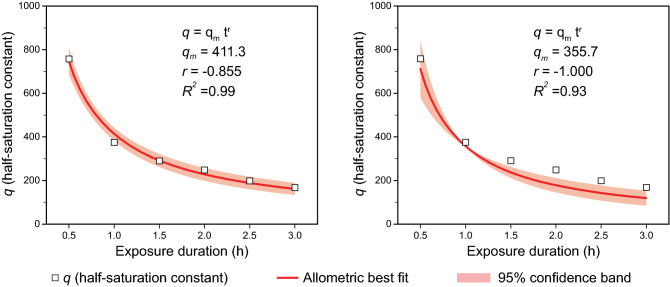
Optimized half-saturation function (left) where *q*_*m*_ = 411.3 and *r* = − 0.855, as well as a simpler half-saturation function (right), consistent with the original model by Rea et al.^17^, where *q*_*m*_ = 355.7 and *r* = − 1.000; *q*_*m*_ is the same as the original model and *r* was simply obviated.

Subsequent modeling combining Eqs. 3 and 4 resulted in optimized values of *q*_*m*_ = 411.3 and *r* = − 0.855 (Eq. 5). The left panel in Fig. 5 shows the functional relationship between exposure duration (in hours) and the optimized half-saturation values, *q*, from Fig. 4.

5 $${CS}_{t}=0.7*\left[1-\frac{1}{1+{\left(\frac{{CL}_{A}}{411.3*{t}^{-0.855}}\right)}^{1.1026}}\right]$$

where,

*CS*_*t*_ corresponds to the absolute response of the SCN as characterized by nocturnal melatonin suppression following a light exposure duration of *t* in hours.

The goal of the present study was to investigate whether the CS function can be simply supplemented by adding light exposure duration (*t*) as an independent stimulus parameter. Since there was no significant difference between the CS formulation based upon a 1-h exposure and the 1-h suppression data from Nagare, et al.^25^ (Table 1), the optimized function (CS_t_, Eq. 5) was simplified. In the original CS model (i.e., based on 1-h exposure), *q* = 355.7, so in the simplified Eq. 6, *q*_*m*_ becomes a constant, *q*_*m*_ = 355.7, and to obviate the exponent, r, entirely *r* = − 1.0. Thus,

6 $${CS}_{t}=0.7*\left[1-\frac{1}{1+{\left(\frac{{CL}_{A}}{355.7*{t}^{-1}}\right)}^{1.1026}}\right]$$

Figure 5 illustrates the optimized half-saturation function, where *q*_*m*_ = 411.3 and *r* = − 0.855, and the simplified half-saturation function, where *q*_*m*_ = 355.7 and *r* = − 1.000. In the simplified CS_t_ function (Eq. 6), CL_A_ and *t* are the only unknowns.

**Figure 6:**
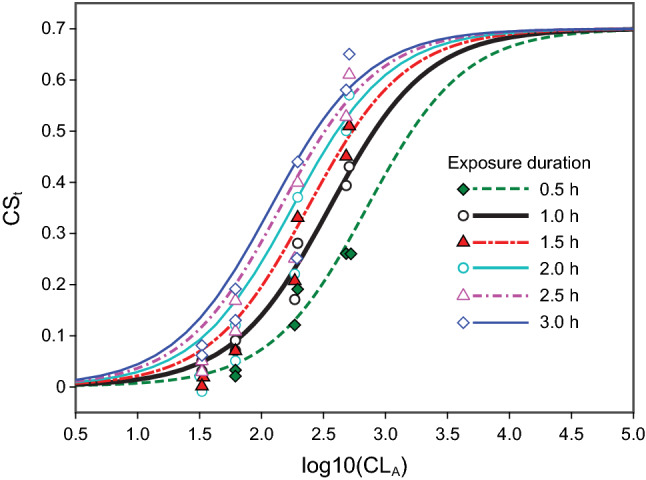
All the data from Nagare et al.^25^ together with simplified CS_t_ functions for the six light exposure durations (*t* = 0.5 to 3.0). The solid black line represents the original CS function based upon 1-h exposure (*t* = 1.0). As can be readily appreciated, it is important to augment the CS formulation (Eq. 6) to include a dynamic factor for duration of light exposure to predict absolute melatonin suppression.

Absolute predictions based upon the proposed CS_t_ function (Eq. 6) are depicted in Fig. 6 for the six durations, from *t* = 0.5 h to *t* = 3.0 h.

### Validation

To validate the simplified function, it was possible to compare the predicted CS_t_ values with nocturnal melatonin suppression data from 11 published studies^28–38^ (Supplementary Tables S2 and S3).

**Figure 7:**
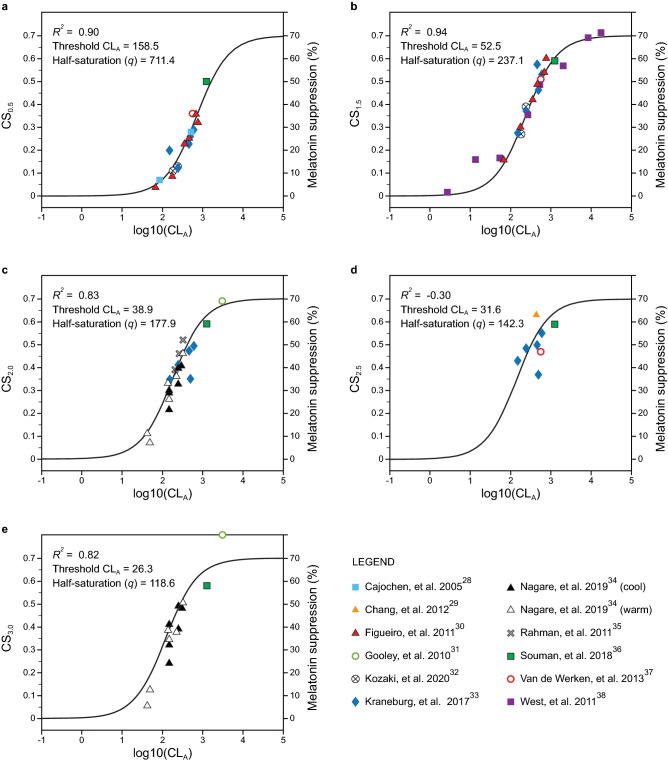
Simplified CS_t_ function predictions for measured or calculated melatonin suppression for exposure durations of 0.5 h (**a**), 1.5 h (**b**), 2.0 h (**c**), 2.5 h (**d**), and 3.0 h (**e**). As discussed in the Supplementary Material, since the 1-h data were used to estimate CS, the data for this exposure duration could not be legitimately included in the validation exercise. Threshold CL_A_ corresponds to circadian light levels estimated to induce nocturnal melatonin suppression of 10%.

**Figure 8:**
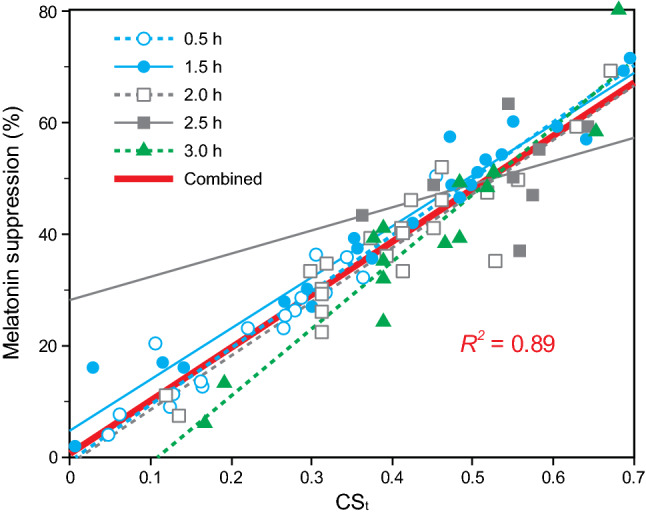
The relationship between the physical stimulus (Φ), measured in terms of CS_t_, and the measured melatonin suppression responses (ψ) from the validation studies in Supplementary Table S3. The symbols correspond to melatonin suppression datasets at the various exposure durations. The lines for each exposure duration correspond to the best fitting linear trends across the respective datasets, whereas the continuous (solid red) line depicts the trend across the five datasets combined. Ignoring the data set for 2.5 h (see text for “Discussion” of this duration), the *R*^2^ value was 0.91.

Figure 7 shows the calculated melatonin suppression data from the selected studies along with a priori predictions from the simplified function (Eq. 6; see Fig. 6). All the curves fit the melatonin suppression data significantly (*p* < 0.001; Supplementary Table S4). Except for the 2.5-h exposure duration, the goodness of fits, R^2^, were greater than 0.80. For the 2.5-h dataset, there simply were not enough data to produce statistically reliable estimates of nocturnal melatonin suppression. Nevertheless, the CS_t_ predictions for 2.5 h go through the center of the melatonin suppression data and, in addition, the fits for the flanking 2.0-h and 3.0-h data were well predicted by the simplified function. These two pieces of indirect supporting evidence suggest that the simplified function is reliable for predicting nocturnal melatonin for the 2.5-h duration. Figure 8 shows the relationship between CS_t_ (now in the physical domain, Φ) and melatonin suppression for the data used in the validation exercise. Taken together, the validation exercise using independent data supports the utility of simplified function, CS_t_, for predicting melatonin suppression during the early biological night.

## Discussion

In the present study we showed that the original CS formulation proposed by Rea et al.^17,18,24^ could be used to predict the amount of nocturnal melatonin suppression during the early biological night for different durations of exposure by adding just one additional duration-dependent parameter, *t*. These findings indirectly support the inference that CL_A_ and CS are robust specifications of the instantaneous luminous stimulus for the circadian system in terms of both spectrum and amount. To predict the amount of nocturnal melatonin suppression, however, CL_A_ and CS are only part of the stimulus specification. As is well known, and shown here, the duration of exposure must also be specified. The simplified CS_t_ formulation was not only useful for predicting the data from Nagare et al.^25^ but provided excellent explanatory power for several other published studies. Thus, specification of the luminous stimulus for suppressing nocturnal melatonin during the early biological night can be described in terms of CS_t_ which itself is defined in terms of the spectrum (CL_A_), the amount (CS), and the exposure duration (*t*) of the luminous stimulus.

The implications of the simplified function, CS_t_, are perhaps most relevant to light exposures in residences prior to sleeping. It is important that evening light not disrupt the circadian system, both in terms of delaying circadian phase and attenuating melatonin synthesis. In that regard, it had been suggested by Rea and Figueiro^39^ that most residential lighting would not produce sufficient light exposures (for typical spectra, amounts, and durations in residences) to significantly suppress melatonin synthesis. Specifically, Rea and Figueiro suggested, as a stated conservative threshold, that people at home in the evening should limit their light exposures to “white” light to 30 lx at the eyes for 30 min. The laboratory study by Nagare et al.^25^ provided a more precise estimate of exposure threshold, suggesting that light exposures to white light in residences should be limited to 50 lx at the eye for 2 h. These suggested exposure limits rely, first, on an assumption about the threshold for light-induced nocturnal melatonin suppression (≈ 10%) and, second, on empirical measurements and observations of lighting in residences in North America and Europe.

A nocturnal melatonin suppression exposure threshold of 10% was chosen for two reasons. First, the 10% value appears to be a good indicator of the “toe” of the logistic function relating log CL_A_ to nocturnal melatonin suppression (e.g., Fig. 4) and, second, because the uncertainty is approximately 10% in melatonin measurements using radioimmune assay methods^39^.

Several studies have reported the amount of light, usually in photopic illuminance (lx), that were or might be incident on the corneas of occupants in their residents. A study by Burgess and Eastman^40^ reported a mean light exposure of 33.0 lx (SD 13.8) over 4 h prior to bedtime, as measured using pendant actiwatches. Scheuermaier et al.^41^ reported a mean light exposure of 34.8 lx (SD 24.1) prior to bedtime (19:00–00:00) for healthy young and older adults, measured using wrist worn actiwatches. In an extensive study involving 72 female school teachers, Rea, et al.^42^ reported mean evening residential vertical light levels of 28 lx, recorded using headband-Daysimeters^27^ between civil twilight and bedtime.

**Figure 9:**
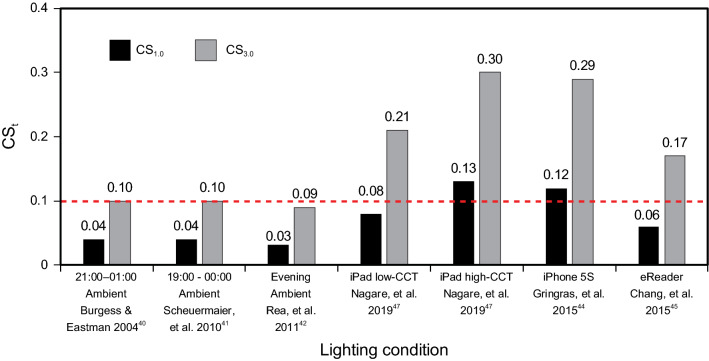
CS_t_ predictions prior to bedtime with typical ambient lighting in residences and with self-luminous devices for durations of 1 h and 3 h. The dashed red line depicts the proposed threshold for activation of the human circadian system.

Warm incandescent, CFL, or LED sources of approximately 2,700 K dominate the residential lighting market^43^. According to the simplified function, CS_t_, a photopic illuminance of 34 lx from both the Burgess and Eastman and the Scheuermaier et al. studies from “warm” sources translates into CS_1.0_ = 0.04 for a 1-h exposure and CS_3.0_ = 0.10 for a 3-h exposure (Fig. 9). A photopic illuminance of 28 lx from the Rea et al. study translates into CS_1.0_ = 0.03 and CS_3.0_ = 0.09.

In modern homes, however, it is probably common today for occupants to experience prolonged light exposures from self-luminous displays. For instance, Gringras et al.^44^ reported that smartphones (iPhone 5S) operated from a typical reading distance (22.5 cm) can deliver a light level of 51 lx at the eye. For typical self-luminous spectra, this would translate into CS_1.0_ = 0.12 and CS_3.0_ = 0.29 (Fig. 9). Chang et al.^45^ recorded an average photopic light level of 32 lx at the eye (n = 12) from eReaders, which translates into CS_1.0_ = 0.06 and CS_3.0_ = 0.17 (Fig. 9). In a more extensive study of self-luminous tablets following up on the earlier work by Wood et al.^46^, Nagare et al.^47^ reported that iPads deliver around 70 lx at the eye for an average viewing distance of 30.5 cm; this translates into CS_1.0_ = 0.13 and CS_3.0_ = 0.30. Using the “Night-shift” setting for the same tablets, CS_1.0_ = 0.08 and CS_3.0_ = 0.21, respectively. In general, Fig. 9 shows that evening ambient light exposures in residences are typically below the proposed threshold of CS_t_ = 0.10, even after 3 h. For self-luminous displays that might be used in the home, however, predicted CS_t_ levels are well above the proposed threshold, even when using the “Night-shift” setting^47^.

Cautions associated with the simplified CS_t_ function deserve mention. Although the present study supports the assumption that the spectral and absolute sensitivities of the SCN are well characterized by CL_A_ and CS, respectively, it should not be assumed that the duration term, *t*, is applicable to melatonin suppression at every time of night. The dataset from Nagare et al.^25^ from which the simplified function was developed, were collected at clock times when control night melatonin levels were increasing. Thus, until further research is completed, the simplified function should only be applicable for predicting nocturnal melatonin suppression on the rising part of the melatonin curve. In this regard, Phillips et al.^48^ have shown that prolonged exposures to light prior to predicted DLMO attenuate the impact of light-induced melatonin suppression at night and, moreover, their particular protocol adds significant variance among subjects to the threshold for melatonin suppression. Perhaps a more obvious caution, the duration term in the simplified function should not be used to predict light-induced phase shifts, during the night or during the day. Even though CS_t_ may be a good representation of the light stimulus to the SCN, light-induced phase shifting responses may not have the same functional relationship to the light stimulus as light-induced nocturnal melatonin suppressions. For example, recent studies have shown that light-induced nocturnal melatonin suppression is different than light-induced phase response^49,50^. Therefore, more research is needed to model the potentially interactive phase response characteristics of the SCN to combinations of spectrum, amount, and duration.

## Conclusions

The present study extended the Rea et al. model by introducing a duration-dependent parameter and a proposed simplified formulation, CS_t_, to predict nocturnal melatonin suppression during the early biological night. Data from a variety of published studies supported quantitative CS_t_ model predictions of nocturnal melatonin suppression. Overall the simplified CS_t_ formulation should be helpful in setting guidelines to limit melatonin suppression for residential applications where people are exposed to light prior to sleeping.

## Supplementary information

Supplementary Information.

## Data Availability

The data that support the findings of this study are available from the corresponding author upon reasonable request.
