# Supplementary material for: Predictions of melatonin suppression during the early biological night and their implications for residential light exposures prior to sleeping
Source: Sci Rep. 2020 Aug 24;10:14114. doi: 10.1038/s41598-020-70619-5 (PMC7445277; doi:10.1038/s41598-020-70619-5)
Supplement: Supplementary file 1 — Supplementary Information. [file 41598_2020_70619_MOESM1_ESM.pdf]

Supplementary Material

Predictions of melatonin suppression during the early biological night and their implications for residential light exposures prior to sleeping

Mark S. Rea, Rohan M. Nagare, Mariana G. Figueiro
Lighting Research Center, Rensselaer Polytechnic Institute, Troy, New York

Methods

Supplementary Table S1. *Post hoc statistical analysis showing that the measured nocturnal melatonin suppression from Nagare, et al. [1] and effective CS did not differ significantly (p >0.05).*

Correlated color temperature (K)	CS level	*t*-test result
2700	0.07	t_{34} = -0.77, p = 0.45
2700	0.14	t_{34} = -0.46, p = 0.65
2700	0.30	t_{31} = -1.08, p = 0.29
2700	0.50	t_{31} = -0.37, p = 0.71
6500	0.07	t_{34} = 0.10, p = 0.92
6500	0.14	t_{30} = 0.00, p = 1.00
6500	0.30	t_{32} = 1.55, p = 0.13
6500	0.50	t_{34} = 0.63, p = 0.54

Results — Validation

The criteria for inclusion of studies in the validation exercise are listed in Supplementary Table S2.

Supplementary Table S2. *Inclusion criteria for studies employed in the model validation.*

Criterion	Description
Participant selection	Healthy adults with no reported ocular dysfunction<br>Reported regular schedule with no trans-meridian travel for at least one week
Intervention	Specified light exposure starting early biological night (19:30 ≤ light pulse start ≤ 02:00)
A control condition	Periodic measurements during dim or dark nighttime conditions
Outcome measure	Melatonin concentrations for suppression estimates
Data for the 1-h reference condition	Melatonin concentration measurements following a 1-h exposure to estimate the circadian stimulus (CS)

Brief overviews of the studies included in the validation exercise are provided in Supplementary Table S3. Some of these studies calculated melatonin suppression (e.g., area under the curve, or AUC) using a different method than that employed by Nagare, et al. [1]. Moreover, the data points may not have been available in the public domain, so the plots relating the light stimulus to

melatonin concentrations or suppression were digitized using a freely available online tool (WebPlotDigitizer ver. 4.2, Ankit Rohatgi, San Francisco, California, USA) and point estimates for different exposure durations were determined.

From these estimates, and to be consistent with Nagare, et al. [1], melatonin concentrations at each sampling time point were normalized to the melatonin value for the first sample taken under that condition. Melatonin suppression was then determined by comparing the normalized melatonin levels collected during the dim light control condition to the normalized levels collected at the corresponding time for each lighting intervention using the following formula:

$$Percent\ suppression = 1 - \left(\frac{Mn}{Md}\right) \times 100 \qquad \text{(S1)}$$

where M_n is the normalized melatonin concentration at a given time point following a light exposure and M_d is the normalized melatonin concentration at the same time point during the control night.

Corneal light exposures were not monitored in most validation studies (e.g., with Daysimeters like those used in the Nagare et al. study). To use those results in the validation exercise, it was necessary to estimate the CS from the reported or calculated melatonin suppression values associated with the 1-h reference condition on which the Rea et al. model is based. This "back-calculated" $CS_{1.0}$ value was then used as the light stimulus for the 0.5-h, 1.5-h, 2.0-h, 2.5-h and 3.0-h melatonin suppression estimates. (Since the 1-h data were used to estimate CS, the data for this exposure duration could not be legitimately included in the validation exercise.) For each of the 5 datasets, a nonlinear least squares fitting analysis based upon the Levenberg-Marquardt [2,3] was applied using curve fitting software OriginPro 2020 (OriginLab Corporation, Northampton, MA, USA) to determine if the *a priori* predicted responses from the simplified function could reliably estimate the validation data ($p < 0.05$).

Supplementary Table S3. *Studies included in the validation exercise for the simplified function.*

Study	Experimental design
Cajochen, et al. [4]	Melatonin suppression was determined using 10 male subjects exposed to 2 h (21:30 – 23:30) of narrowband (460 nm or 555 nm) light, both at approximately 2.8 x 10^{13} photons cm^{-2} s^{-1}<br>Some of these data could not be used because melatonin concentrations did not rise in the control condition after 1 h.
Chang, et al. [5]	Melatonin suppression was determined using 39 healthy subjects exposed to a 4100 K fluorescent light source (10,000 lx) for different durations (0.2, 1.0, 2.5, 4.0 h), all centered at approximately 02:00
Figueiro, et al. [6]	Melatonin suppression was determined using 9 older adults exposed to 1.5-h (00:00-01:30) of narrowband light (470-nm) at varying irradiances (0.7 to 72 μW cm^{-2})
Gooley, et al. [7]	Melatonin suppression was determined using 66 healthy subjects exposed to 6.5-h (23:00-05:30) of continuous narrowband short-wavelength (460 nm) or longer-wavelength (555 nm) light at varying irradiances

Study	Experimental design
	Only the data made public by the authors for 2 representative subjects exposed to a dose of 12.85 log photons cm^{-2} s^{-1} could be used. Data for the 555 nm condition could not be included in the validation exercise because calculated 1-h melatonin suppression was zero.
Kozaki, et al. [8]	Melatonin suppression was determined using 15 male subjects exposed to 1.5-h (01:00 – 02:30) of photon density matched flickering and non-flickering blue light (~ 14 lx)
Kraneburg, et al. [9]	Melatonin suppression was determined using 16 healthy subjects exposed for 4-h (22:00 – 02:00) to 7 spectrally distinct polychromatic white light sources (CCTs: 1600 K – 14,000 K) delivering 200 lx at the eye<br>Some of these data could not be used because melatonin concentrations did not rise in the control condition after 2.5 h. Calculated 1-h melatonin suppression values for 2 light sources from Kraneburg et al. (2017) (CCTs: 1600 K, 1950 K) were both zero, and hence, no data points could be included for these spectra.
Nagare, et al. [10]	Melatonin suppression was determined using 12 adults and 12 adolescents exposed to 4-h (23:00 – 03:00) of polychromatic white light (2700 K, 5600 K) delivering a CS of 0.25 at the eye
Rahman, et al. [11]	Melatonin suppression was determined using 12 adult subjects exposed to 12-h (20:00 – 08:00) of polychromatic light after completely filtering light at wavelengths < 480 nm, wavelengths < 460 nm, or partially filtering light at wavelengths < 480 nm<br>Data for one condition (100% filter < 480 nm) could not be included in the validation exercise because calculated 1-h melatonin suppression was zero.
Souman, et al. [12]	Melatonin suppression was determined using 15 healthy subjects exposed to 3-h (starting 2 h prior to habitual bedtime) of white light (2 sources with high or low radiant power in μW cm^{-2} between 450 – 500 nm) matched for photopic illuminance of 175 lx<br>Data for one condition (low power between 450 – 500 nm) could not be included in the validation exercise as calculated 1-h melatonin suppression was zero.
Van de Werken, et al. [13]	Melatonin suppression was determined using 33 male subjects exposed to 8-h (23:00 – 07:00) of short-wavelength attenuated polychromatic white light (193 lx), or full-spectrum light (256 lx)<br>Data for one condition (short-wavelength attenuated light) could not be included in the validation exercise because calculated 1-h melatonin suppression was zero.
West, et al. [14]	Melatonin suppression was determined using 8 adult subjects exposed to 1.5-h (02:00 – 03:30) of short-wavelength light (469 nm) at varying corneal irradiances (0.1 to 600 μW cm^{-2})

Supplementary Table S4. *Curve-fitting results for the melatonin suppression data analyzed from the 11 studies listed in Supplementary Table S3 and shown in main article Fig. 7.*

Exposure duration	Curve-fitting results
0.5 h	$F_{1,16} = 749.8$, $p < 0.001$
1.5 h	$F_{1,22} = 2415.6$, $p < 0.001$
2.0 h	$F_{1,21} = 1016.9$, $p < 0.001$
2.5 h	$F_{2,6} = 106.9$, $p < 0.001$
3.0 h	$F_{1,13} = 407.9$, $p < 0.001$

References

1 Nagare, R., Rea, M. S., Plitnick, B. & Figueiro, M. G. Nocturnal melatonin suppression by adolescents and adults for different levels, spectra, and durations of light exposure. *J. Biol. Rhythms* **34**, 178-194, doi:10.1177/0748730419828056 (2019).

2 Marquardt, D. W. An algorithm for least-squares estimation of nonlinear parameters. *J. Soc. Ind. Appl. Math.* **11**, 431-441, doi:10.1137/0111030 (1963).

3 Levenberg, K. A method for the solution of certain non-linear problems in least squares. *Q. Appl. Math.* **2**, 164-168, doi:10.1090/qam/10666 (1944).

4 Cajochen, C. *et al.* High sensitivity of human melatonin, alertness, thermoregulation, and heart rate to short wavelength light. *J. Clin. Endocrinol. Metab.* **90**, 1311-1316, doi:10.1210/jc.2004-0957 (2005).

5 Chang, A. M. *et al.* Human responses to bright light of different durations. *J. Physiol* **590**, 3103-3112, doi:10.1113/jphysiol.2011.226555 (2012).

6 Figueiro, M. G. *et al.* The impact of self-luminous electronic devices on melatonin suppression. *Dig Tech Pap* **42**, 408-411, doi:10.1889/1.3621337 (2011).

7 Gooley, J. J. *et al.* Spectral responses of the human circadian system depend on the irradiance and duration of exposure to light. *Sci. Transl. Med.* **2**, 31ra33, doi:10.1126/scitranslmed.3000741 (2010).

8 Kozaki, T., Hidaka, Y., Takakura, J. Y. & Kusano, Y. Salivary melatonin suppression under 100-Hz flickering blue light and non-flickering blue light conditions. *Neurosci. Lett.* **722**, 134857, doi:10.1016/j.neulet.2020.134857 (2020).

9 Kraneburg, A., Franke, S., Methling, R. & Griefahn, B. Effect of color temperature on melatonin production for illumination of working environments. *Appl. Ergon.* **58**, 446-453, doi:https://doi.org/10.1016/j.apergo.2016.08.006 (2017).

10 Nagare, R., Plitnick, B. & Figueiro, M. G. Effect of exposure duration and light spectra on nighttime melatonin suppression in adolescents and adults. *Light. Res. Technol.* **51**, 530-540, doi:10.1177/1477153518763003 (2019).

11 Rahman, S. A., Marcu, S., Shapiro, C. M., Brown, T. J. & Casper, R. F. Spectral modulation attenuates molecular, endocrine, and neurobehavioral disruption induced by

nocturnal light exposure. *Am. J. Physiol. Endocrinol. Metabol.* **300**, E518-527, doi:10.1152/ajpendo.00597.2010 (2011).

12 Souman, J. L. *et al.* Spectral tuning of white light allows for strong reduction in melatonin suppression without changing illumination level or color temperature. *J. Biol. Rhythms* **33**, 420-431, doi:10.1177/0748730418784041 (2018).

13 Van de Werken, M., Gimenez, M. C., de Vries, B., Beersma, D. G. & Gordijn, M. C. Short-wavelength attenuated polychromatic white light during work at night: limited melatonin suppression without substantial decline of alertness. *Chronobiol. Int.* **30**, 843-854, doi:10.3109/07420528.2013.773440 (2013).

14 West, K. E. *et al.* Blue light from light-emitting diodes elicits a dose-dependent suppression of melatonin in humans. *J. Appl. Physiol.* **110**, 619-626, doi:10.1152/japplphysiol.01413.2009 (2011).
